# A case and literature review of axial spondyloarthritis and immunoglobulin A vasculitis: Rare association or under-recognized?

**DOI:** 10.1177/2050313X251341130

**Published:** 2025-05-24

**Authors:** Robin Sia, Mueed Mian

**Affiliations:** 1 Department of Rheumatology, The Northern Hospital, Epping, Victoria, Australia; 2 Faculty of Medicine and Health, University of Sydney, Camperdown, New South Wales, Australia; 3 Epping Specialist Group, Epping, Victoria, Australia

**Keywords:** axial spondyloarthritis, ankylosing spondylitis, IgA vasculitis, Henoch–Schönlein purpura, IgA nephropathy

## Abstract

Axial spondyloarthritis (axSpA) is a chronic inflammatory arthritis affecting the spine and sacroiliac joints, often accompanied by extra-musculoskeletal manifestations involving the eyes, gut, and skin. Other organ systems, including the heart (aortic insufficiency), lungs (upper-lobe predominant interstitial fibrosis), and kidneys (nephritic syndrome), may also be affected. Immunoglobulin A vasculitis (IgAV), formerly known as Henoch–Schönlein purpura, is the most common systemic vasculitis in children and is typically self-limited. It is characterized by palpable purpura, arthritis or arthralgia, abdominal pain, and renal involvement. Studies suggest a potential link between elevated serum immunoglobulin A levels and active inflammation in axial spondyloarthritis. Here, we present a case of a Caucasian male diagnosed with immunoglobulin A vasculitis, leading to the identification of previously unrecognized axial spondyloarthritis. In addition, we reviewed the current literature on IgAV occurring in patients with axial spondyloarthritis.

## Introduction

Axial spondyloarthritis (axSpA) is a chronic inflammatory disease involving the axial skeleton and can also cause peripheral manifestations such as asymmetrical oligoarthritis, enthesitis, as well as dactylitis. Although the association between seronegative spondyloarthropathies and immunoglobulin A (IgA) nephropathy has been previously documented, the association with IgA vasculitis (IgAV) remains unclear, with only 10 reported cases found in our literature review. Furthermore, among the cases found, axSpA with negative HLA-B27 and IgAV is extremely rare, with only three reported cases. This article discusses a case of newly diagnosed IgAV with an incidental diagnosis of HLA-B27-negative radiographic axSpA (r-axSpA) in a gentleman, as well as to review the current literature highlighting the relationship between these two conditions.

## Case description

**Figure 1.**
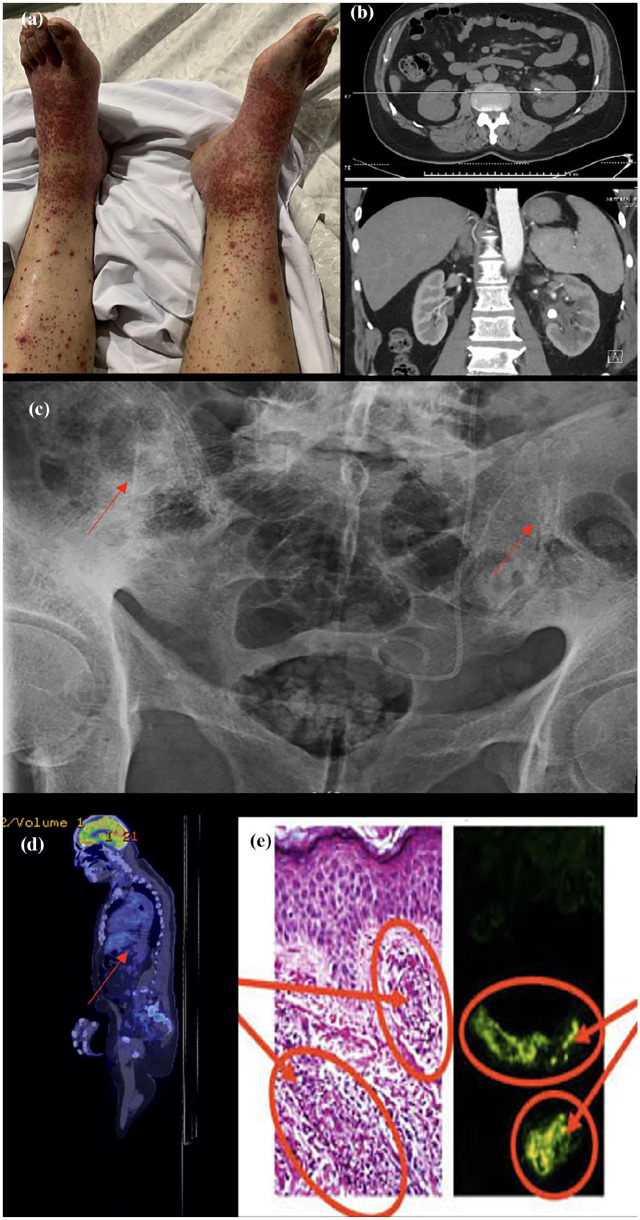
(a) Non-blanching petechiae on the patient’s bilateral distal lower limbs, (b) CT-IVP which showed moderate left hydroureteronephrosis secondary to a 10 mm obstructing calculus at the left pelviureteric junction, and associated urothelial thickening, enhancement, and adjacent stranding, (c) X-ray of SIJ demonstrating bilateral sacroiliitis estimated at grade 4 on the right and grades 2–3 on the left, (d) FDG-PET scan showing an incidental mass highly suspicious for a GIST with lack of FDG avidity. (e) Histopathology of skin biopsy with the left image demonstrating leukocytoclastic vasculitis with a positive immunofluorescence stain of IgA on the right. CT-IVP: CT-intravenous pyelography; FDG-PET: fludeoxyglucose-18 positron emission tomography; GIST: gastrointestinal stromal tumour; IgA: immunoglobulin A; SIJ: sacroiliac joint.

We report the case of a man in his 50s who presented with abdominal and testicular pain, accompanied by haematuria. His symptoms were preceded by a bilateral purpuric rash on the ankles and distal hands, which had appeared 4 days earlier. The rash was palpable but neither painful nor pruritic (Figure 1(a)). There were no recent upper respiratory tract infections or fevers nor any recent medication changes. In regards to the scrotal tenderness and swelling, there was no dysuria, discharge, or recent trauma. On examination, his vital signs were within normal limits. On further examination, there was a non-blanching palpable purpuric rash noted at his distal legs and proximal arm. His testicular exam demonstrates a swollen and tender right testicle.

Blood tests demonstrated a normal full blood count as well as liver function tests and kidney function. His C-reactive protein (CRP), urine albumin–creatinine ratio, and urine protein–creatinine ratio were elevated with no glomerular red cells (Table 1). His urine culture and sensitivity showed *Staphylococcus aureus*, however, given he was systemically well with no infective prodrome or signs of bacteraemia, this was thought to be a contaminant by the infectious disease team and was not treated.

**Table 1.** Patient’s lab investigations.

Investigation	Value	Normal range
Haemoglobin (g/L)	124	115–155
White cell count (×10^9^/L)	8.2	4.0–12.0
Platelet count (×10^9^/L)	174	150–400
eGFR	>90	>90
Creatinine (µmol/L)	78	45–90
Urea (mmol/L)	7.5	3.5–8.0
Sodium (mmol/L)	136	135–145
Potassium (mmol/L)	4.1	3.5–5.2
ALP (U/L)	67	30–110
GGT (U/L)	35	<37
ALT (U/L)	33	5–35
AST (U/L)	30	<31
CRP (mg/L)	94	<6
uACR (mg/mmol)	26.6	<2.5
uPCR (mg/mmol)	37	<30
ESR (mm/h)	62	<21
CK (µ/L)	51	30–150
ANA	1:160 (speckled)	<1:80
IgA (g/L)	10.6	0.6–4.6
IgG (g/L)	16.9	7.0–16.5
IgM (g/L)	0.7	0.4–2.7
ANCA	Negative	
RF	2	<20 U/mL
ACPA	3	<20 U/mL
HLA-B27	Negative	–

eGFR: estimated glomerular filtration rate; ALP: alkaline phosphatase; GGT: gamma glutamyl transferase; ALT: alanine aminotransferase; AST: aspartate aminotransferase; CRP: C-reactive protein; uACR: urine albumin–creatinine ratio; uPCR: urine protein–creatinine ratio; ESR: erythrocyte sedimentation rate; CK: creatine kinase; ANA: anti-nuclear antibody; IgA: immunoglobulin A; IgG: immunoglobulin G; IgM: immunoglobulin M; ANCA: antineutrophil cytoplasmic antibodies; RF: rheumatoid factor; ACPA: anti-citrulinated peptide antibody.

Further imaging was done including a scrotal ultrasound which confirmed right-sided epididymitis with a moderate hydrocele, a computerized tomography intravenous pyelography (CT-IVP) which showed moderate left hydroureteronephrosis secondary to a 10 mm obstructing calculus at the left pelviureteric junction, and associated urothelial thickening, enhancement, and adjacent stranding, with an incidental fusion of the sacroiliac joints (SIJs) bilaterally (Figure 1(b)). An X-ray of his SIJs was subsequently performed, which demonstrated bilateral sacroiliitis estimated at grade 4 on the right and grade 3 on the left (Figure 1(c)).

Following his incidental finding of bilateral sacroiliitis, a targeted history revealed that he has had ongoing inflammatory-sounding lower back pain over the past 10 years with minimal peripheral joint involvement. His examination further demonstrates an occiput-to-wall distance of 1 cm, modified Schober’s test of 3 cm, with restriction on frontal and lateral spinal flexion, with mild SIJ tenderness on flexion, abduction, and external rotation more so on his right than his left SIJ. His Bath AS Disease Activity Index score, which measures his degree of axSpA, was 1.9 points, which demonstrated low disease activity.^1^ There were no extra-articular manifestations at the time of our examination. Using the Assessment of Spondyloarthritis International Society criteria, using the radiological arm, he fulfils the criteria of axSpA with evidence of sacroiliitis on his imaging and at least one other symptom to support this, which is his response to non-steroidal anti-inflammatory drugs (NSAIDs) and also his history of anterior uveitis.^2^

An autoimmune screen, including HLA-B27 testing, was done, which was found to be negative (Table 1). A positron emission tomography scan was done in the setting of his constellation of diseases and positive findings demonstrated an incidental mass with a lack of fludeoxyglucose avidity highly suspicious for a gastrointestinal stromal tumour (Figure 1(d)). A skin biopsy of his rash was done, which demonstrated leukocytoclastic vasculitis (LCV) with a positive immunohistochemistry stain of IgA (Figure 1(e)). A diagnosis of IgAV was subsequently made.

He was started on prednisone monotherapy at 25 mg with a gradual tapering regimen. Following discharge, he continues to be monitored in outpatient rheumatology, where his axSpA remains stable, and there have been no further IgAV relapses despite discontinuation of prednisolone therapy.

## Discussion

Axial spondyloarthritis (axSpA) is a chronic inflammatory disease with a predilection involving the axial skeleton and encompasses both radiographic (r-axSpA) and non-radiographic axSpA.^3^ The primary site of involvement of axSpA is the enthesis and the subchondral bone.^4,5^ This leads to axial inflammation and bone destruction as well as new bone formation driven by tumour necrosis factor-α and IL-23/IL-17.^5^ HLA-B27 is a genetic marker strongly associated with axSpA. Approximately 80%–90% of patients with axSpA test positive for HLA-B27, although its presence is not necessary for diagnosis. The exact role of HLA-B27 in disease pathogenesis remains under investigation, but it is believed to contribute to immune dysregulation, misfolded protein accumulation, and an abnormal inflammatory response.^6^ However, about 10%–20% of those with axSpA are HLA-B27 negative. The prevalence of HLA-B27-negative disease is higher in certain populations, such as those of African or Middle Eastern descent, and can present atypically, such as later onset of disease and lower genetic predisposition.^6^

There has been some documented increase in IgA levels both in serum and in the skin of patients with axSpA. The mean serum IgA was 38% higher in patients with axSpA compared to controls.^7^ The increase in IgA levels was associated with systemic inflammation, especially subclinical gut inflammation, suggesting a link between mucosal immune activation and the pathogenesis.^7^ There have only been 10 reported cases of axSpA associated with IgAV. The following table includes all the reported cases of IgAV and concomitant axSpA, with the majority of cases being HLA-B27 positive (Table 2). Our case is unique, as his HLA-B27 testing returned negative.

**Table 2.** Characteristics of cases reported in the literature of patients with concomitant IgAV and axSpA.

Authors	Year	Sex	Age (years)	HLA-B27 status	Active axSpA
Peeters et al.^8^	1990	Male	35	Negative	No
Peeters et al.^8^	1990	Male	50	Negative	No
Beauvais et al.^9^	1993	Male	50	Negative	Yes
Beauvais et al.^9^	1993	Male	45	Positive	Yes
Machet et al.^10^	1997	Male	31	Positive	Yes
Kobak et al.^11^	2014	Male	26	Positive	Yes
John et al.^12^	2019	Male	26	Positive	Yes
Kamath et al.^13^	2022	Male	40	Positive	No
Demouveaux et al.^14^	2024	Male	25	Positive	No
Demouveaux et al.^14^	2024	Male	30	Positive	No

axSpA: axial spondyloarthritis; IgAV: immunoglobulin A vasculitis.

The first two cases documented by Peeters et al. discuss a new diagnosis of IgAV in patients with inflammatory bowel disease (IBD) and axSpA, with immunofluorescence studies showing perivascular deposition of IgA in the skin biopsies of both patients as well as in the renal mesangium of one of the patients.^8^ Furthermore, IgA immune complexes were found in the serum samples of the patients.^8^ Skin changes are not characteristic of axSpA compared to IBD, which include erythema nodosum, erythema multiforme, pyoderma gangrenosum, psoriasis, nodular necrobiosis and epidermolysis bullosa, and rarely cutaneous polyarteritis nodosa and granulomatous vasculitis.^8^ These cases highlight the possibility of IgA as a possible pathogenesis. Furthermore, it has been proposed that the concept of abnormal IgA secretion via microbial antigenic stimulation of the digestive mucosa plays a role in the pathogenesis of AS.^7^ IgAV can often be diagnosed with skin biopsies demonstrating LCV with IgA deposits. Leukocytoclastic vasculitis (LCV) also termed hypersensitivity vasculitis, is a form of small-vessel vasculitis and can be associated with autoimmune conditions, drugs (penicillin, sulfonamides, NSAIDs, thiazides, retinoids, and quinolones), and infections. Kobak et al. support the fact that axSpA can be one of the causes of LCV and that treatment of this condition relies on treating the cause.^11^

The observation of elevated IgA levels in patients with axSpA has prompted further research into the potential role of IgA antibodies in early disease detection.^15^ In addition, serum IgA levels have clinical significance in several aspects. Notably, regular treatment with NSAIDs has been shown to reduce serum IgA levels, suggesting a potential disease-modifying effect of NSAIDs in axSpA. In the future, serum IgA levels could serve as a complementary biomarker for monitoring disease activity alongside erythrocyte sedimentation rate and CRP in clinical practice.

## Conclusion

This case report and literature review emphasize the rare association between axSpA and IgAV. The relationship between these two diseases is still unclear. However, evidence have demonstrated that given the elevated levels of IgA in patients with axSpA, there may be a higher risk of developing IgAV. Therefore, clinicians should have a low threshold to recognize and diagnose IgAV in patients with a background history of axSpA. Early recognition of IgAV is essential as renal involvement may occur.

## Learning points

- This case report and literature review emphasize the rare association between axial spondyloarthritis (axSpA) and immunoglobulin A vasculitis (IgAV) and although the relationship between these two conditions is still unclear, evidence have demonstrated that given the elevated levels of IgA in patients with axSpA, there may be a higher risk to develop IgAV.
- Clinicians should have a low threshold to recognize and diagnose IgAV in patients with a background history of axSpA. Early recognition of IgAV is essential as renal involvement may occur.
- There may be a role for the use of therapeutics that may target both conditions, especially given the limitations in treatment options for IgAV.
